# Low-dose lipopolysaccharide inducing continuous and obvious increase in urinary protein in hyperglycemic rats and the underlying mechanism

**DOI:** 10.1371/journal.pone.0288876

**Published:** 2023-07-19

**Authors:** Mulan Wang, Qin Gong, Chenliang Zha, Song Xu, Dong Yu, Tianyu Huang, Yulin Feng, Hong Sun, Jun Li

**Affiliations:** 1 Department of Pharmacy, State Key Laboratory of Innovative Drugs and Efficient Energy-Saving Pharmaceutical Equipment, Nanchang, China; 2 Department of Pharmacy, Jiangxi University of Traditional Chinese Medicine, Nanchang, China; 3 Department of spleen and stomach, The Affiliated Hospital of Jiangxi University of Chinese Medicine, Nanchang, China; 4 Institute of Medicinal Plant, Chinese Academy of Medical Sciences and Peking Union College, Beijing, China; Indiana University Purdue University at Indianapolis, UNITED STATES

## Abstract

Proteinuria is an important hallmark of diabetic nephropathy models, however it takes a long time for the proteinuria and is not stable. Therefore, low-dose lipopolysaccharide (LPS) was investigated in this work to induce rapid and stable proteinuria in hyperglycemic rats and the underlying mechanism was studied. Hyperglycemia rats was induced by high-fat feeding combined with intraperitoneal injection of streptozotocin (STZ). After 21 days, the model rats received a subinjury dose of 0.8 mg / kg LPS intraperitoneally (i.p.). We detected related biochemical indexes at different time periods after LPS injection and examined the expression of glomerular podocyte-associated proteins. Simultaneously, we measured expression of inflammatory factors, apoptotic proteins and albumin (ALB) in the renal cortex and renal medulla, respectively. PAS (Periodic Acid Schiff) staining was used to observe renal pathology. After LPS injection, urinary microalbumin (umALB) increased significantly and lasted longer. The expression of Nephrin, Podocin and necroptosis factor kappa B (NF-κB) in rennal cortex and Interleukin 18 (IL-18), Caspase-1, NF-κB and ALB in the renal medulla was significantly changed. Pathologically, the glomerular basement membrane was observed to be significantly thickened, the renal tubules were dilated, and the epithelial cells fell off in a circle. LPS promoted the continuous increase in urinary microalbumin in hyperglycemic rats, which was related to the damage to the glomerular basement membrane and renal tubular epithelial cells and to the inflammatory reaction in the kidney involved in NF-κB signaling, and this pathological damage can help to establish a stable model of diabetic nephropathy with increased proteinuria.

## 1 Introduction

Urinary protein is an important clinical feature of diabetic nephropathy that reflects the pathophysiological changes of glomerular filtration and tubular reabsorption [1–4]. In drug research, detecting changes in total protein and microalbumin in urine is also an important pharmacodynamic index to evaluate the effect of drugs on diabetic nephropathy. Simultaneously, the level of urinary protein is also an important indicator to evaluate the animal model of diabetic nephropathy. However, hyperglycemia combined with low dose LPS-induced proteinuria has little been reported [5]. The renal lesions in animals caused by simple hyperglycemia were relatively mild [6, 7], and the urine protein rose more slowly and for less time. Therefore, based on the establishment of a hyperglycemia model by high-fat diet and STZ injection, this study explored the feasibility of the model of promoting renal injury and persistent proteinuria by injection of LPS. On the basis of the rat model [8, 9], LPS injection was added to aggravate renal injury, and the changes in urinary protein in different periods after LPS injection were systematically observed. At the same time, the underlying mechanism of the continuous increase in urinary protein was explored from the aspects of glomerular basement membrane, inflammation and apoptosis to promote research on the rat model of diabetic kidney injury, and this information will be helpful for the application of related drug research.

## 2 Materials and methods

### 2.1 Animals and housing conditions

Male Sprague Dawley (SD) rats weighing 180 ~ 200 g were purchased from Hunan SJA Laboratory Animal Co., Ltd. (Changsha, China), [SCXK (Xiang) 2016–0002]. Humane care was given according to the 3R principles of applying laboratory animals. Feeding and sterile surgery experiments were completed in the Laboratory of Barrier Environment of the Jiangxi Bencao-Tiangong Technology Co., Ltd. (Nanchang, China), [SYXK (Gan) 2018–0002]. The animal room was maintained at 23 ± 2°C and 40% - 60% humidity with a daily 12-hour light/dark cycle. Both feed and water were provided ad libitum. This experimental research protocol was approved by the Institutional Animal Care & Use Committee of Jiangxi University of Traditional Chinese Medicine (TCM) and the Animal Welfare & Ethics Committee of Jiangxi University of TCM (JZLLSC20230009). The experimental procedure strictly followed the relevant guidelines of the Experimental Animal Welfare and Ethics of China.

### 2.2 Reagents

The following reagents were used: Streptozotocin (STZ, Abmole, Shanghai, China), Lipopolysaccharide (LPS, Sigma, Shanghai, China), Biochemical kit (BUN(blood urea nitrogen), CRE(creatinine), AST(aspartate amino-transferase), ALT (alanine amino-transferase), CSF(total protein), Beijing Leadman Biochemical Co., Ltd. Beijing, China; mALB (microalbumin), Mike Bio Co., Ltd. Chengdu, China), Blood glucose test strips (Roche, Shanghai, China), Hifair III 1st strand cDNA Synthesis SuperMix for qPCR (YEASEN, Shanghai, China), Hieff qPCR SYBR Green Master Mix (YEASEN,Shanghai, China), TRIzol, (Beijing Jumei Biotechnology Co., Ltd., Beijing, China), 5× protein loading buffer (Solarbio, Beijing, China), Glycogen PAS staining kit (LEAGENE, Beijing, China), RIPA Lysis Buffer (Strong) (CWBIO, Jiangsu, China).

### 2.3 Experimental procedure

Of 35 SD rats, 10 were reserved as the normal group and the LPS group (5 rats in each group), and the other 25 were used as hyperglycemia models. The normal group and LPS group were fed a normal maintenance diet, while the model group was fed a high-fat diet (Guangdong Provincial Medical Laboratory Animal Center, Foshan, China) for 5 days, fasted overnight, and injected intraperitoneally with streptozotocin (STZ; in 0.05 M citrate buffer; pH 4.5;) at 60 mg / kg [10, 11]. After 2 hours of injection, they resumed eating. After 5 days, the fasting blood glucose was measured, and the rats with blood glucose values in the range of 16.7 mmol—30 mmol were selected for grouping into the model group and the model + LPS group, with 7~9 rats in each group. After 20 days of modeling, overnight urine was collected to detect total protein (CSF)and microalbumin (mALB) in urine. On the 21st day, the normal + LPS group and the model + LPS group were intraperitoneally injected with 0.8 mg / kg LPS, and the normal control group and the model control group were intraperitoneally injected with an equal volume of normal saline.

In order to dynamically study the pathological changes after LPS administration, the tested samples were taken and determined at different times: At day 6 and day 10 after LPS injection, the animals were fasted at 9:00 am, and blood was collected from the tail vein at 3:00 pm to detect blood glucose, separately. At day 7 and day 11 after LPS injection and before LPS administration, the rat urine was collected for the urinary protein test, respectively. On the 9th and 12th days after LPS injection, rats were anesthetized with isoflurane, blood was collected from the orbital veins for biochemical index detection. After the blood collected at day12, the rats were anesthetized with isoflurane and then killed by cervical dislocation, the kidneys were isolated. The right kidneys were stored at -80°C for mRNA and protein expression and the left kidneys were fixed in 4% formaldehyde for morphological detection.

### 2.4 Blood glucose and biochemical index detection

The blood glucose was determined with blood glucose test strips

Urine and serum were collected as previously described [12] for the detection of urinary protein and liver and kidney function. Overnight urine was collected, the urine volume was accurately measured, the total urine protein (uCSF) and urine microalbumin (umALB) were detected by a 7100 automatic biochemical analyzer (Hitachi, Japan), and finally, by multiplying by urine volume, total urinary protein and microalbumin were obtained. The liver and kidney function was evaluated by the index of ALT, AST, BUN and CRE. All the samples in serum were detected according to the kid instructions by using the 7100 automatic biochemical analyzer.

### 2.5 mRNA expression analysis

mRNA expression was analyzed using real-time PCR as previously described [13]. The renal cortex and renal medulla were isolated from rat kidney. Total RNA was extracted from the renal cortex and renal medulla using an RNA extraction kit (TRIzol) and reverse-transcribed to cDNA using the Hifair III 1st strand cDNA Synthesis SuperMix for qPCR according to the manufacturer’s instructions. Real-time PCR analysis of specific genes was performed on a 7500 Real-Time PCR system (Applied Biosystems, USA) using Hieff qPCR SYBR Green master mix. The primers were designed by GenBank NCBI (https://www.ncbi.nlm.nih.gov/) and synthesized by Nanjing GenScript Biotechnology Co., Ltd. The rat primers were as follows: *β-actin*, sense: 5’-CTCTGTGTGGATTGGTGGCT-3’, antisense: 5’-GCTCAGTAACAGTCCGCCT-3’; *Nephrin*, sense: 5’-TAGTTATTAATAGATTGTCAGGAGTCTGTCAC-3’, antisense: 5’-CCGCTCGAGCGGAGAAGCCAGGAGTTCAGATTT-3’; *Podocin*, sense: 5’-TAGTTATTAATGCACTAAACGGGAAGGAAT-3’, antisense: 5’-GGAAGATCTTCCTTGCCTTCTTGTCATCCCT-3’; and *Alb*, sense: 5’-GTGAGCGAGAAGGTCACCAA-3’, antisense: 5’-TTTCACCAGCTCAGCGAGAG-3’. *β*-actin served as an internal control.

### 2.6 Protein expression analysis

Protein expression was analyzed using Western blotting as previously described [14]. Primary antibodies against nephrin (rabbit monoclonal antibody, ab216341), Caspase-3 (mouse monoclonal antibody, ab13585), and IL-6 (mouse monoclonal antibody, ab9324) were purchased from Abcam (USA). Podocin (rabbit polyclonal antibody, TA351459), NLRP3 (rabbit polyclonal antibody, TA336895), IL-18 (rabbit polyclonal antibody, TA324190), Caspase-1 (rabbit polyclonal antibody, TA323204), and NF-κΒ (rabbit polyclonal antibody, TA890002) antibodies were purchased from ORIGENE (USA). ALB antibody (rabbit polyclonal antibody, 16475-1-AP) was purchased from Proteintech (China). Goat anti-mouse IgG-HRP (ZB2305) and goat anti-rabbit IgGHRP (ZB2301) secondary antibodies were purchased from Zhongshan Jinqiao Biotech-Company (Beijing, China). The targeted proteins were visualized with the Super Signal West Femto Chemiluminescent Substrate (Thermo Scientific Pierce, Beijing, China), and the intensities of the visualized bands were analyzed using Quantity One software (Bio-Rad at Shanghai, China). *β*-actin (mouse monoclonal antibody, TA-09, Zhongshan Jinqiao Biotech company, Beijing, China) was used as an internal control. The data are expressed as the ratio to *β*-actin.

### 2.7 Morphological analysis of kidney

The kidney-tissue samples were prepared according to our previous literature method [15] and the Glycogen PAS staining kit instructions for PAS staining of kidney tissue. The tissues were observed under a light microscope (Olympus, Japan). The histopathological diagnoses were performed independently by two different research scientists. All images were recorded at 400 × amplification.

### 2.8 Data analysis

All values are expressed as the mean ± S.E.M., independently. All results were analyzed using one-way analysis of variance (ANOVA) with F value determination. The F test was performed using GraphPad Prism 8.01 software (GraphPad, San Diego, California, USA). Student’s *t test* was performed between two groups after the F test. A P value below 0.05 was considered statistically significant. The statistical graphs were produced using GraphPad Prism 8.01 software as described above.

## 3 Results

### 3.1 Changes in the protein in urine

**Fig 1:**
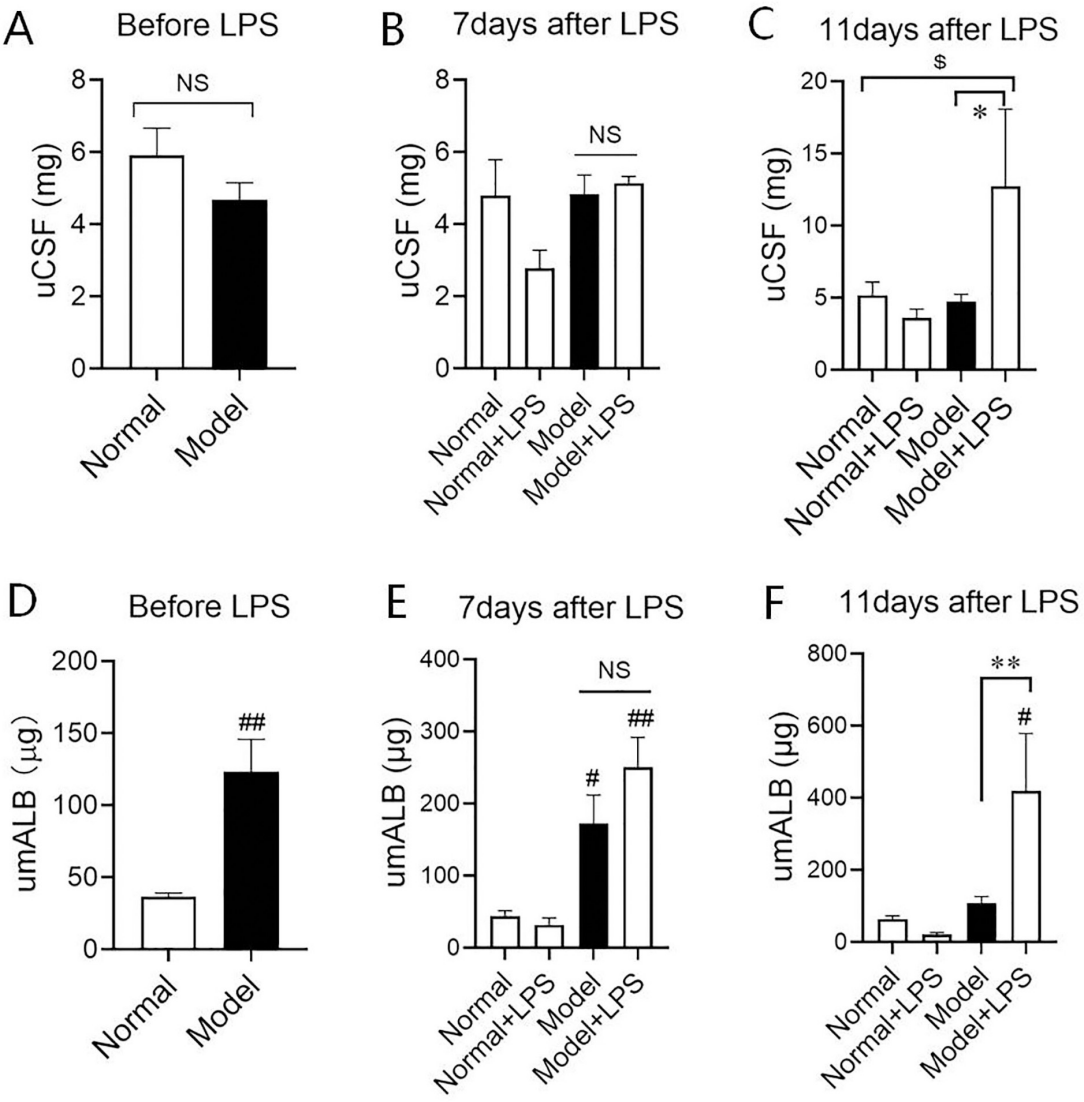
Changes of urinary total protein (uCSF) and urinary microalbumin (umALB) in hyperglycemia rats before and after the intraperitoneal injection of LPS. (A)—(C) Changes of uCSF before and after LPS injection. (D)—(F) Changes of umALB before and after LPS injection. The data were shown as mean ± S.E.M from 3~14 rats in each group. # and ## compared with the normal groups, *P* < 0.05 and *P* < 0.01. * and ** compared with model groups, *P* < 0.05 and *P* < 0.01. $ compared with normal groups, P = 0.057. NS: no significance.

Urinary protein is a key index reflecting kidney injury, thus it’s often used for judging whether the renal damage during the diseases including diabetes. In our experiments, we found the urinary total protein (uCSF) of the hyperglycemic rats showed no significant difference from that of the normal group, after establishing the hyperglycemia rat model and before injecting LPS (Fig 1A), but the urine microalbumin (umALB) was increased significantly compared with that of the normal group (Fig 1D). Seven days after the injection of LPS, umALB in both the model group and model + LPS group was significantly increased compared with that in the normal group, and the increase in the model + LPS group was higher than that in the model group in a tendency (Fig 1B and 1E), but the total protein of urine in both model and model + LPS groups were not increase distinctively compared with that of normal groups. Eleven days after LPS injection, umALB in model groups was not increased continuously compared with normal groups indicating urinary protein in only hyperglycermia could not stay in a high level. At the same time, the uCSF of the model + LPS group showed a continuous increase and was higher than that of the model group but in a up-tendency compared with that of the normal group (*P* = 0.057) (Fig 1C), and the umALB of the model + LPS group increased significantly compared with both the normal group and the model group (Fig 1F), implying that LPS can persistently significantly elevate urinary albumin in hyperglycemia model animals.

### 3.2 Changes in blood glucose and serum biochemical indices

**Fig 2:**
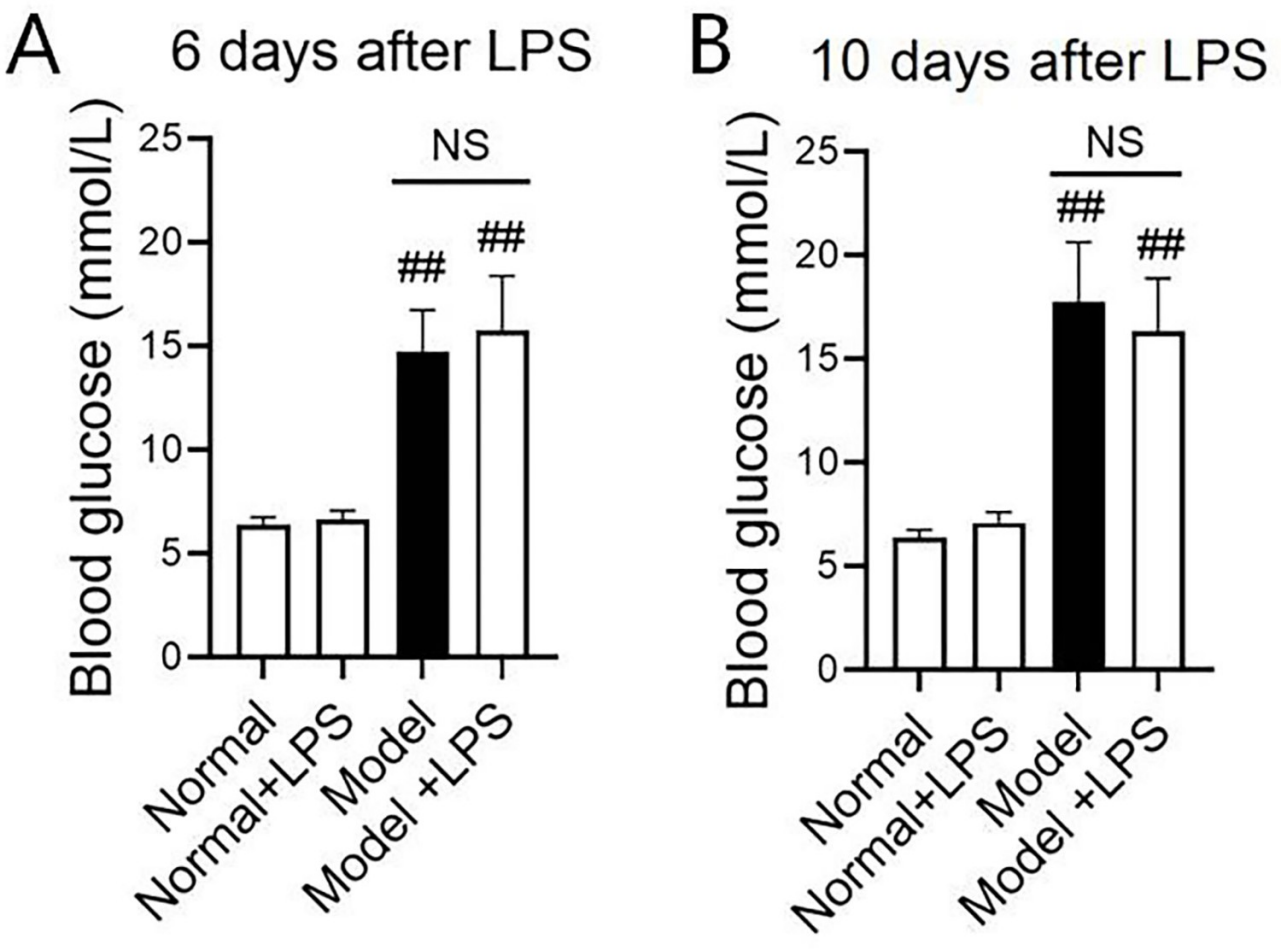
Changes of blood glucose on the 6^th^ and 10^th^ day after the intraperitoneal injection of LPS. (A) Changes of blood glucose on day 6. (B) Changes of blood glucose on day 10. The data were shown as mean ± S.E.M with 5~9 rats in each group. ## compared with the normal group, *P* < 0.01. NS: no significance.

In order to know whether blood glucose was disturbed by LPS, we detected the blood glucose of rats one day before the urinary protein test. The results showed that after 6 days and 10 days of LPS injection, the blood glucose levels of the model group and the model + LPS group were significantly increased compared with that of the normal group (*P* < 0.01), and there was no significant difference between the two model groups (Fig 2), indicating that LPS had no significant effect on the blood glucose of the animals.

**Fig 3:**
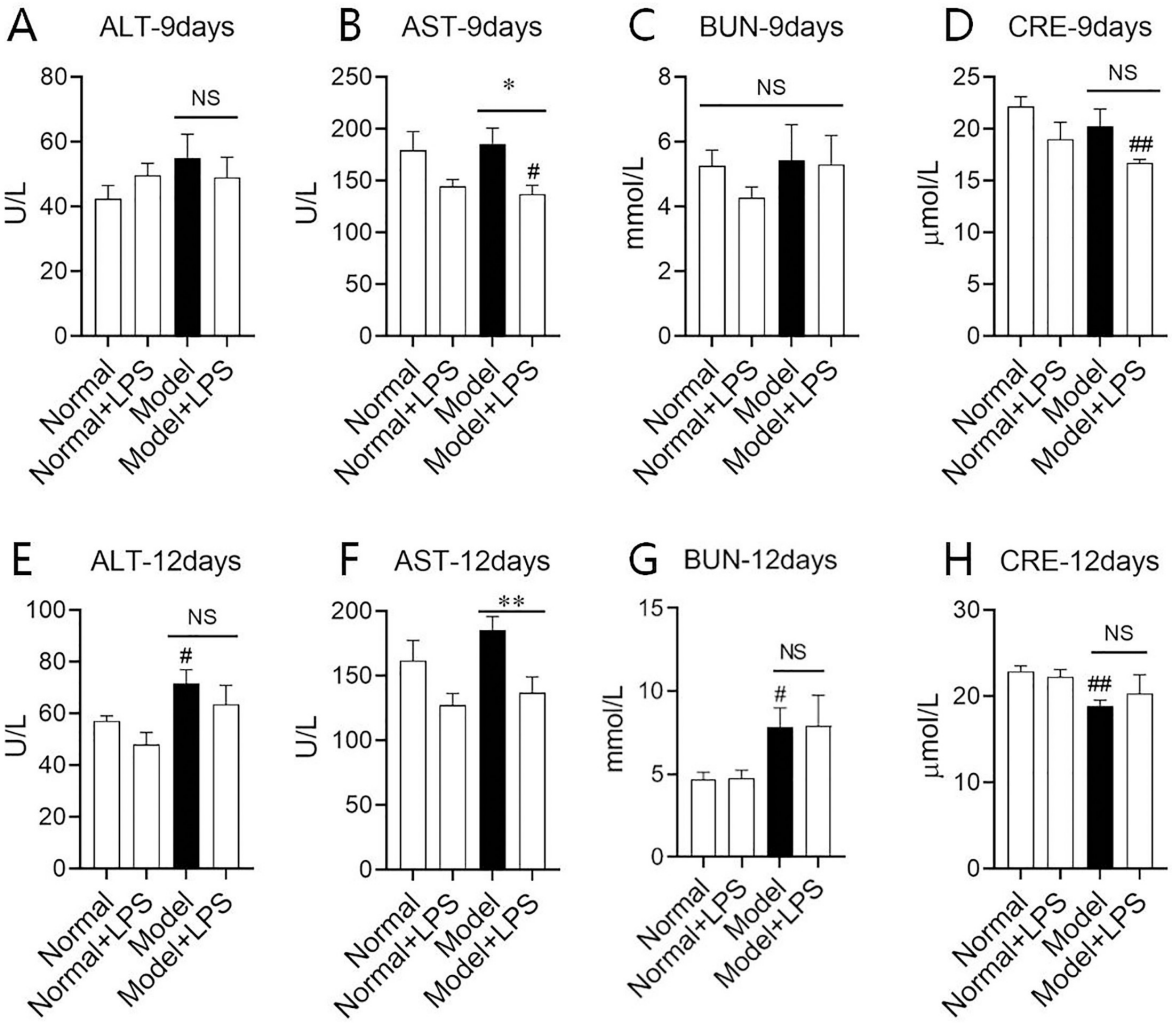
Changes of liver function (AST and ALT) and kidney function (BUN and CRE) on day 9 and day 12 after the intraperitoneal injection of LPS. (A)—(D) Changes of liver function and kidney function on the 9^th^ day. (E)—(H) Changes of liver function and kidney function on the 12^th^ day. The data were shown as mean ± S.E.M with 5~9 rats in each group. # and ##, compared with normal groups, *P* < 0.05 and *P* < 0.01. * and **, compared with model groups, *P* < 0.05 and *P* < 0.01. NS: no significance.

To verifying if LPS in the low dosage could injury the liver and kidney and the underlying mechanism of urinary protein increase, we then detected serum AST and ALT reflecting the liver function, and BUN and CRE reflecting the kidney function. Fig 3 presents the rat liver and kidney function indices after LPS administration. Nine days after LPS injection, AST and CRE in the model + LPS group were significantly lower than those in the normal group, while ALT and BUN did not change significantly. After 12 days of LPS injection, serum ALT and BUN in the model group were significantly higher than those in the normal group (*P* < 0.05) and CRE was significantly lower than that in the normal group (*P* < 0.01), but there was no significant difference between the two model groups indicating LPS of the lower dose did not influence both liver and kidney function.

Taken together, LPS in the low dosage (0.8 mg / kg, i.p.) does not disturb the blood glucose and liver-kidney function.

### 3.3 Changes in Nephrin and Podocin mRNA and protein expression in kidney tissue

**Fig 4:**
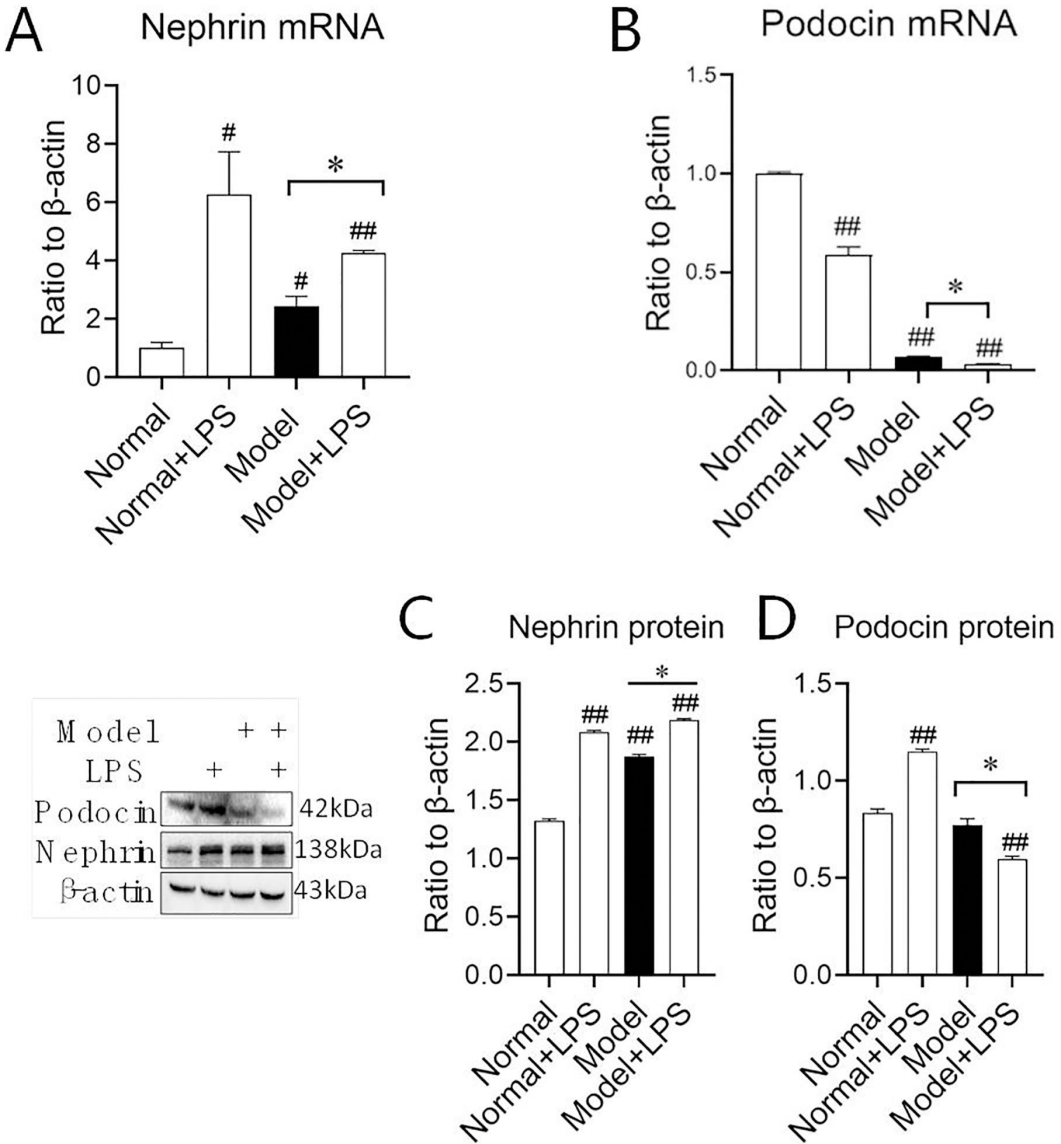
Protein and mRNA expression of Nephrin and Podocin in renal cortex after the intraperitoneal injection of LPS. **(A)–(B)** mRNA expression of Nephrin and Podocin. **(C)–(D)** Protein expression of Nephrin and Podocin. The data were shown as mean ± S.E.M., # and ##, compared with normal groups, *P* < 0.05 and *P* < 0.01. *, compared with model groups, *P* < 0.05. NS: no significance.

Urine protein discharge is related to the increase of glomerular basement membrane permeability. Nephrin and Podocin are two key proteins in the membrane. In order to further explore the mechanism of proteinuria induced by LPS, we conducted mRNA and protein expressions of these two proteins. We found that the mRNA expression of Nephrin in the three groups was significantly upregulated compared with that in the normal group indicating LPS could stimulate Nephrin expression directly (Fig 4A). And, Podocin mRNA expression in three groups was decreased distinctly and there was significant differences between model group and model + LPS groups, suggesting LPS could stimulate Podocin mRNA expression in hyperglycermia (Fig 4B). While in the protein expression, Nephrin was dramatically upregulated in the three groups similar to that of mRNA expression (*P* < 0.01), and the expression in the model + LPS group was higher than that in the model group (*P* < 0.05) (Fig 4C). However, Podocin protein expression in model + LPS groups was apparently decrease compared with the normal and model groups, although the level of Podocin in normal + LPS still increased (*P* < 0.01). Simultaneously, Podocin protein expression in model groups showed no difference (Fig 4D). All that suggests LPS has a certain promoting effect on renal podocyte injury in the hyperglycemia model.

### 3.4 Changes in the protein expression of related inflammatory factors in the renal cortex and renal medulla

We have got, from above, that LPS-induced urinary protein increase was correlated with Podocin expression downregulation. Therefore the inflammatory cytokines, such as NLRP3 signaling, were detected because the inflammation reaction was often correlated to the membrane damage. We isolated the cortex and medulla of the rat kidney for detecting the expression of the proteins because we demand to know the site of the urinary protein discharge induced by LPS exactly.

**Fig 5:**
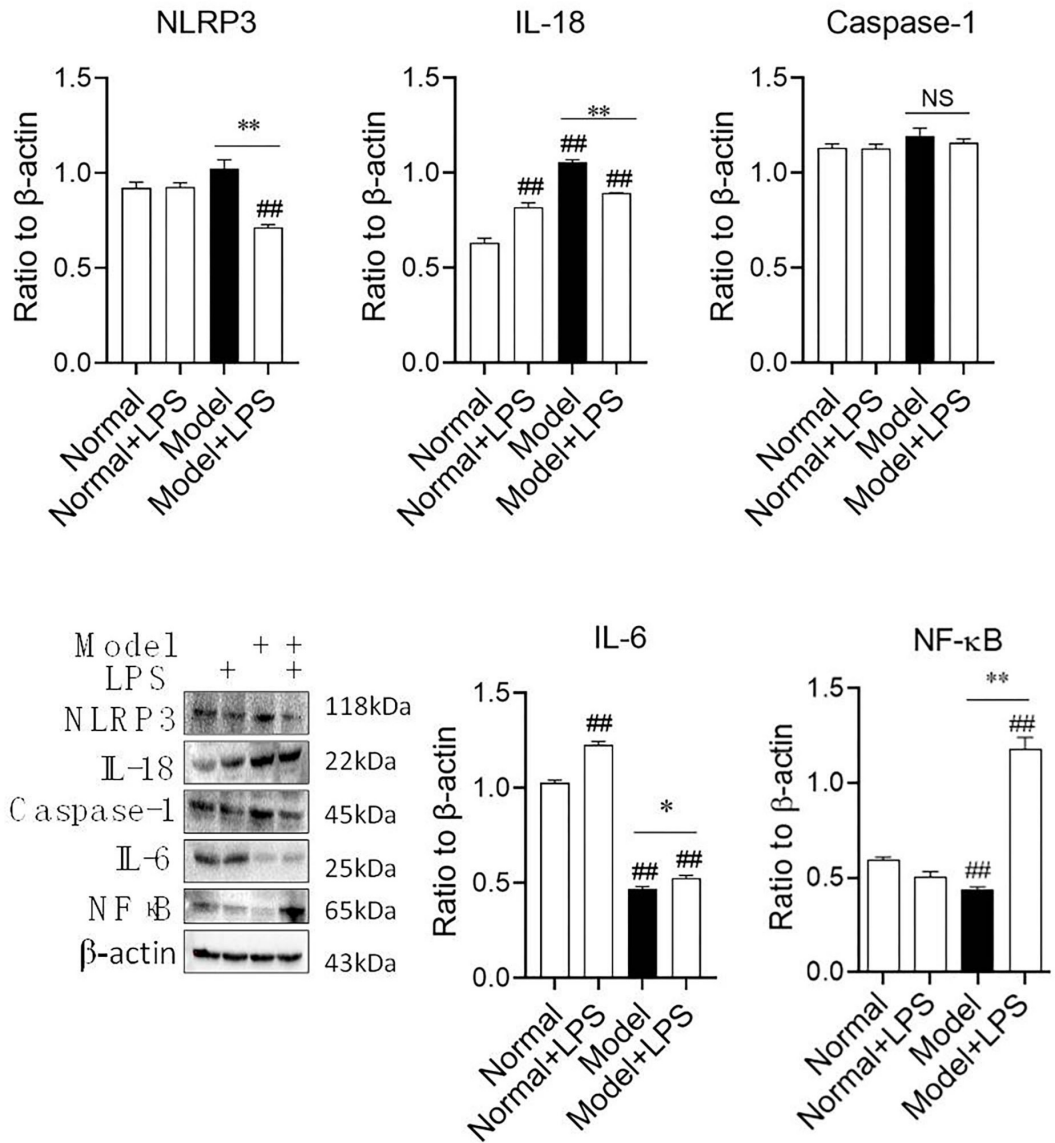
Protein expression of inflammatory cytokines in rat renal cortex after the intraperitoneal injection of LPS. The data were shown as mean ± S.E.M., ##, compared with normal groups, *P* < 0.01. * and **, compared with model groups, *P* < 0.05 and *P* < 0.01. NS: no significance.

In the renal cortex (Fig 5), NF-κB expression in the model + LPS group was obviously upregulated compared with that in the model group. IL-18 was significantly upregulated in the normal + LPS, model, and model + LPS groups, but was not more upregulated after LPS injection in the model + LPS group. Compared with the normal group, NLRP3 was upregulated in the model group but downregulated after LPS injection. LPS alone stimulated the upregulation of IL-6, but in the hyperglycemia model, IL-6 was downregulated. Although IL-6 was upregulated in the model + LPS group, it was still lower than that in the normal group. The above results suggest that LPS mainly stimulates the expression of NF-κΒ in the renal cortex of hyperglycemia model rats without correlation to NLRP3 signaling.

**Fig 6:**
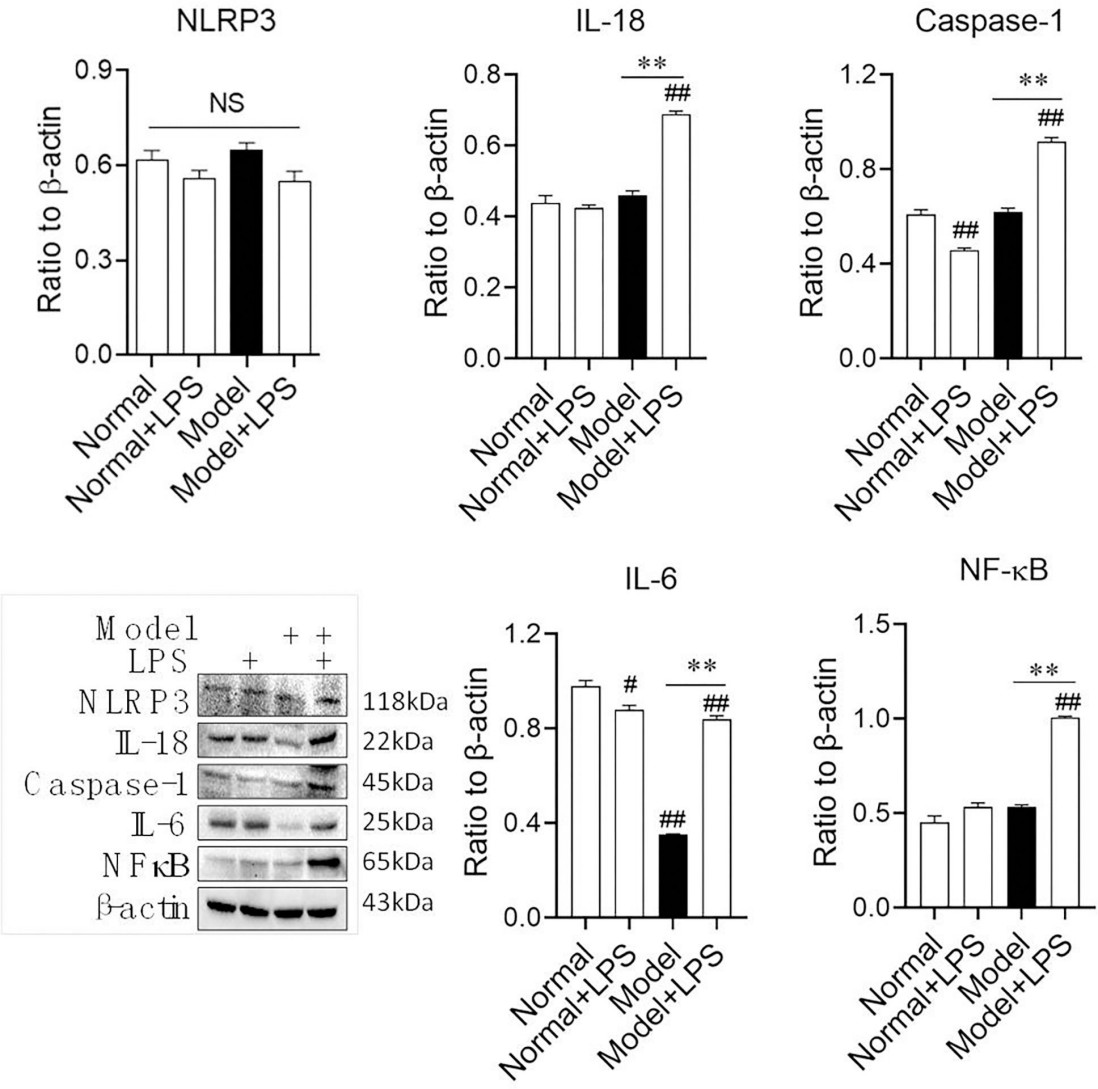
Protein expression of inflammatory cytokines in rat renal medulla after the intraperitoneal injection of LPS. The data were shown as mean ± S.E.M., # and ##, compared with normal groups, *P* < 0.05 and *P* < 0.01. **, compared with model groups, *P* < 0.01.

In the medulla (Fig 6), we found that after LPS injection, inflammatory cytokines, except NLRP3, were dramatically upregulated compared to those in model rats. Although IL-6 was significantly upregulated by LPS injection compared with the model group, it was still downregulated compared with the normal group, which is interesting that IL-6 protein expression downregulated during hyperglycemia. In the renal medulla of hyperglycemic rats, LPS stimulated the expression of inflammatory factors mainly through the IL-18 and caspase-1/NF-κB signaling pathways.

### 3.5 Changes in Caspase-3 and ALB protein expression in kidney tissue

**Fig 7:**
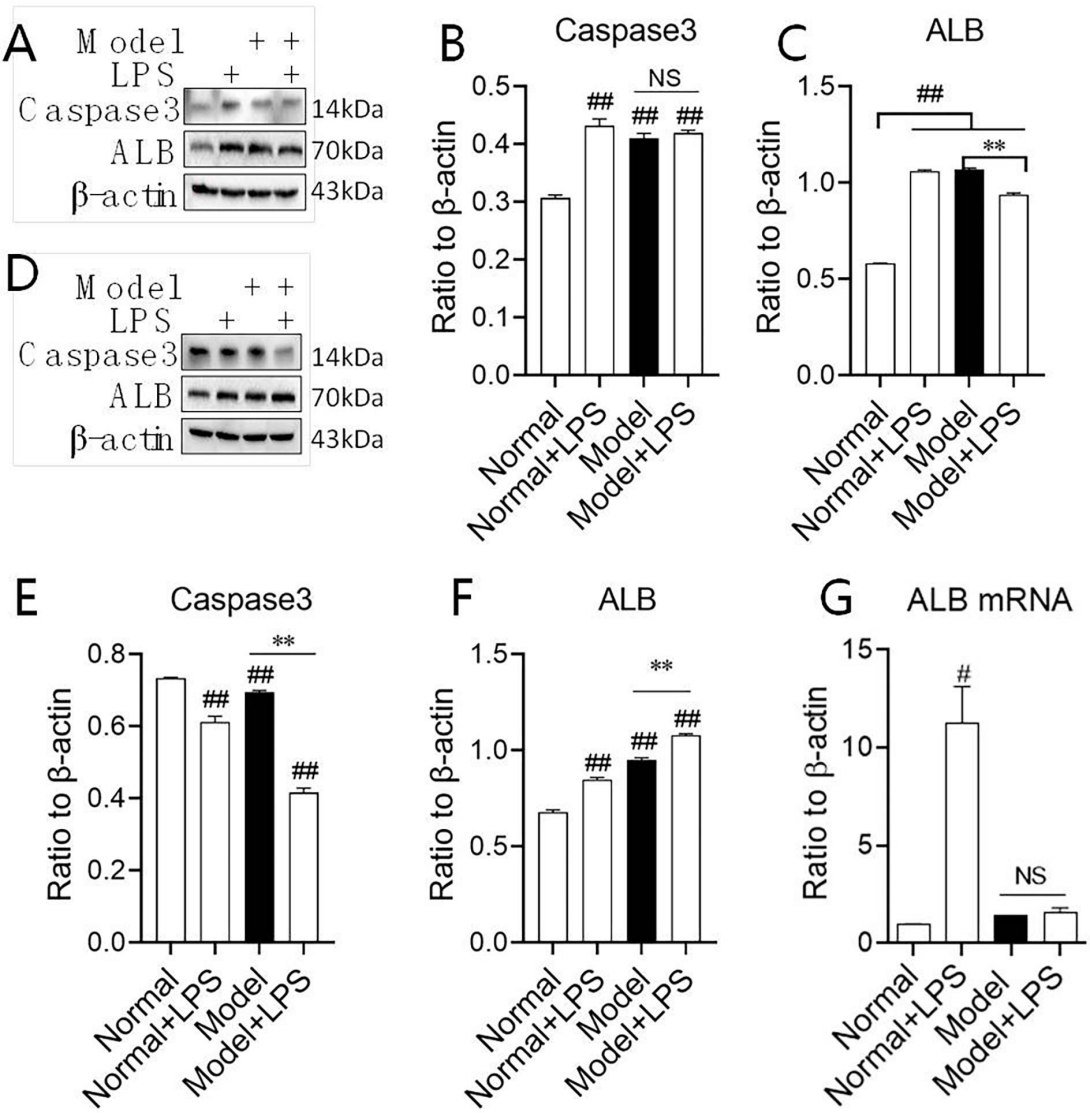
The expression of Caspase3 and ALB in the both renal cortex and medulla after the intraperitoneal injection of LPS. **(A)—(C)** Protein expression of Caspase3 and ALB in the renal cortex. **(D)—(F)** Protein expression of Caspase3 and ALB in the renal medulla. **(G)** mRNA expression of ALB in renal medulla. The data were shown as mean ± S.E.M., # and ##, compared with the normal group, *P* < 0.05 and *P* < 0.01. **, compared with model groups, *P* < 0.01. NS: no significance.

To verifying ALB discharge in renal cortical or/and medulla injury by LPS, we expressed Caspase-3 and ALB in the renal cortex and renal medulla, respectively (Fig 7). In the cortex, the expression of Caspase-3 was upregulated in all three groups compared with the normal group suggesting that there has been the apoptosis by LPS/hyperglycemia (Fig 7A and 7B). However, Caspase-3 was not significantly upregulated after LPS injection during hyperglycemia indicating LPS could not stimulate Caspase-3 upregulation further. In the renal medulla, Caspase-3 was downregulated in all three groups compared with the normal group and even dramatically in the model + LPS group, implying the apoptosis by LPS mainly arose in the cortex (Fig 7D and 7E).

In the renal cortex and renal medulla, ALB expression was dramatically upregulated in all three groups compared with that in the normal group (Fig 7C and 7F). In the cortex, LPS could directly stimulate ALB expression in normal rats, suggesting the membrane was injured by LPS. However, during hyperglycemia LPS could not stimulate ALB expression further and ALB expression decreased slightly compared to the model group, which needs to be studied deeply (Fig 7C).

In the medulla, ALB was more upregulated in the model + LPS group than in the model group. LPS could also stimulate ALB expression in normal groups, but could cause ALB expression in a distinct upregulation in hypoglycemic rats (Fig 7F). Having known to that renal tubules are mostly distributed in renal medulla, ALB protein in the medulla was higher than that in the cortex during the course the protein discharge.

To further understand whether the elevated ALB protein expression in the renal medulla was due to glomerular injury protein escape or the gene expression of ALB in the renal medulla tubules, we detected the expression of ALB mRNA in the renal medulla. The results showed that the expression of ALB mRNA was upregulated in the normal + LPS group while there was no significant change in between the model groups and the model + LPS group, suggesting that LPS stimulated *Alb* expression in renal medulla cells of normal rats but had no effect on hyperglycemia rats (Fig 7G). These results suggest that the increased expression of ALB protein in the renal medulla in model + LPS group was caused by glomerular escape into the renal tubules.

### 3.6 Morphological changes of glomeruli and tubules

**Fig 8:**
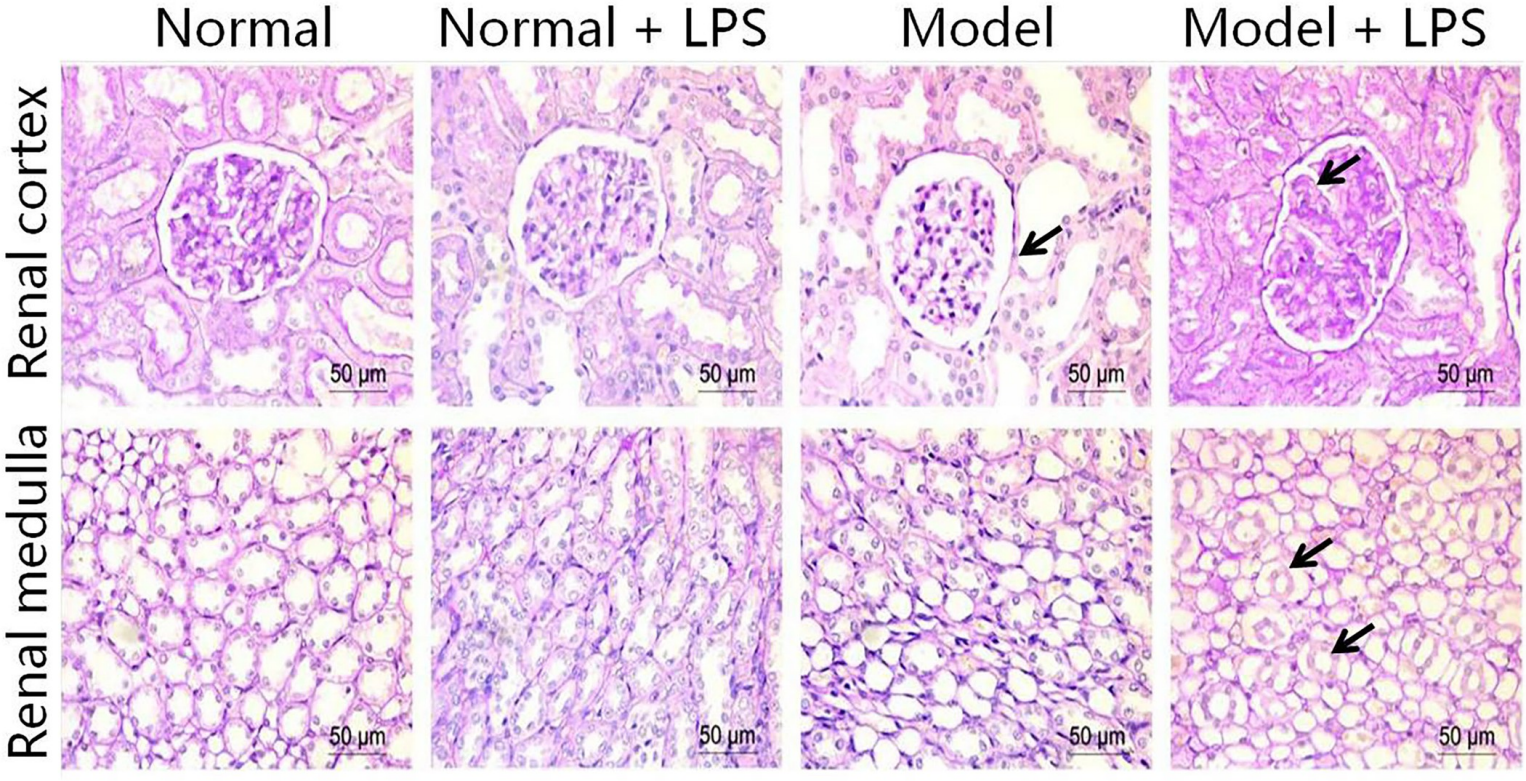
Morphological images of renal cortex and renal medulla cells in each group (400×amplification).

Lastly, we prepared the image of the rat kidney tissue to study the underlying mechanism of LPS inducing protein urine in morphology. Fig 8 shows the pathological changes in the rat kidney. In the normal group, the kidney structure was clear and showed normal glomeruli (renal body), renal tubules, and interstitium. In the LPS group, the structure was still normal, and no obvious damage was found. In the hyperglycemia rats, the glomerular basement membrane was slightly thickened, the renal tubules were dilated, and the epithelial cells were flattened. In the hyperglycemia + LPS group, the glomerular basement membrane was significantly thickened, dilation of the renal tubules was observed, and the epithelial cells fell off in a circle in both the cortex and medulla, supporting that LPS could injure glomeruli and cause proteinuria during the hyperglycemia.

## 4 Discussion

Proteinuria is an important marker of kidney damage [16]. In the diabetes model, the appearance of microalbumin in urine indicates early kidney damage, but the simple hyperglycemia model causes milder kidney damage, with a long model period, and proteinuria is not easy to sustain [6, 7]. In a previous study of acute kidney injury, our research group established a variety of common clinical acute kidney injury models, including an LPS-induced acute kidney injury model in mice [17]. This model mainly stimulated the secretion of tissue inflammatory factors. Current research has shown that the pathogenesis of diabetes is closely related to inflammation [18, 19], therefore, we injected a subinjury dose of LPS into diabetic rats to observe the changes in urinary protein and the related mechanisms that cause this change. The dose of LPS used in our previous studies was mostly 10 mg / kg with that LPS damaged the kidneys. However, if this dosage was used, the mortality of rats can be significantly increased; thus, we screened low-dose LPS through a prelimited experiment, which can cause proteinuria without increasing rat death. We used doses of LPS of 2 mg / kg, 1 mg / kg and 0.8 mg / kg to determine the optimal dose for the experiment. After comprehensive investigation through repeated tests, we finally chose 0.8 mg / kg as the dose to increase proteinuria in hyperglycemic animals without any rat mortality. From the literature [5], high-fat feeding was used for 4 weeks combined with intraperitoneal injection of STZ 40 mg / kg to induce rat hyperglycemia. One week later, diabetic nephropathy (DN) rats with qualified blood glucose were selected to be injected with LPS 1 mg / kg through the tail vein to induce the expression of inflammatory factors in diabetic nephropathy. After 7 weeks of administration, the urine protein, biochemical indices of renal function, apoptosis and inflammatory factors in the model + LPS group were significantly higher than those in the model group. In contrast to this literature report, the model in our experiment was only fed a high-fat diet for 5 days combined with intraperitoneal injection of 60 mg / kg STZ and was injected with LPS 20 days after modeling based on systematic observation of the changes in urinary protein after LPS injection. The time of this model was relatively shortened compared to that of previous models, but the urinary protein was still significantly increased by LPS injection in the hyperglycemia rats, indicating that this experimental model is beneficial for shortening the time of drug evaluation experiments.

The results showed that LPS prolonged the duration of microalbumin in the urine of model animals without affecting the fasting blood glucose level of the animals. Through the detection of liver and kidney function at different time periods after LPS injection, the liver and kidney function of the LPS group alone did not significantly increase compared with the normal group, while the ALT and BUN of the model group and model + LPS group increased to a certain extent, but there was no significant difference between the model + LPS group and the model group, indicating that this small dose of LPS had no significant effect on liver or kidney function.

It has been reported in the literature that gene *Alb* could be expressed in kidney [20]. In order to verify whether proteinuria caused by LPS is caused by the protein leakage of kidney or the expression of *Alb* in kidney, we expressed ALB protein and mRNA in renal cortex and medulla, respectively. The results showed that both LPS and hyperglycemia can cause ALB protein expression in renal cortex and medulla apparently, but LPS can further increase ALB expression in renal medulla of hyperglycemia rats, and this increase has nothing to do with its gene expression, suggesting that should be caused by the protein leakage during kidney damage.

To further understand the reasons for the persistent proteinuria, we examined changes in the Nephrin and Podocin proteins on the glomerular filtration membrane. The kidney glomerulus is a highly specialized structure that ensures the selective ultrafiltration of plasma so that essential proteins are retained in the blood [21]. Attached to the outside of the glomerular basement membrane are the podocyte feet processes, which form the last barrier of the glomerular filtration membrane, and Nephrin and Podocin are important proteins in the cleft [22]. The results of this experiment showed that after injection of LPS, the expression levels of both Nephrin mRNA and protein in each group were significantly upregulated, and the expression of Podocin protein in the model + LPS group was downregulated, suggesting that LPS can promote the damage of glomerular podocytes in hyperglycemia rats. In addition, this abnormally elevated expression of Nephrin may be related to the fact that the loss of podocytes activates a vicious cycle in the glomerulus, in which the remaining podocytes compensate and expand to cover the remaining basement membrane [23], which needs to be further studied in the complex mechanism. Recent studies have shown that local accumulation of a large number of ALBs (such as in the kidney) can cause damage by activating NF-κB and triggering inflammation [24–26]. Also, inflammatory factors can stimulate the upregulation of Nephrin expression, which is consistent with the results of this study. With the abnormal increase in Nephrin, the protein expression of ALB in the kidney was significantly upregulated. However, the urinary protein in both the LPS group and the model group did not continue to increase, suggesting that there may be other mechanisms that need to be studied further.

NF-κB is the most important transcription factor in the pathogenesis of diabetic nephropathy [27], and it can be activated by various stimuli, such as cytokines, oxygen free radicals, inhaled particulate matter, ultraviolet radiation, and bacterial or viral products [28]. In diabetic nephropathy, proteinuria itself is an important activator of NF-κB and an important proinflammatory stimulus for renal tubular cells [29]. NF-κB in the renal cortex and NF-κB, IL-18 and Caspase-1 in the renal medulla of the model + LPS group were apparently upregulated, indicating that LPS can promote inflammatory factors by the transcription factor NF-κB in the hyperglycemia model. However, what is the downstream target of NF-κB during the hyperglycemia? That’s an interesting topic which is needed to be studied further. In addition, we found that the protein expression of IL-6 downregulated distinctly both in the renal cortex and medulla during hyperglycemia. Interestingly, LPS could antagonize IL-6 decline in the medulla but without any effect in the cortex. It is known that IL-6 could promote the inflammatory reaction by attracting the chemokines to the local place for the inflammation [30] reflecting IL-6 relation to the immune function. Our results displayed that IL-6 protein decreased during hyperglycemia implying there had been a poor immune response and LPS stimulated IL-6 expression in the contrary in both the cortex and medulla suggesting the existing of a complex mechanism.

Caspase-3 is a key enzyme and effector molecule in the apoptosis pathway [31]. Activated Caspase-3 can promote the activation of downstream apoptosis-related proteins, resulting in apoptosis [32]. The results showed that LPS alone and hyperglycemia can both induce the activation of Caspase-3, and the protein expression of Caspase-3 in hyperglycemia rats after injection of LPS was not higher than that in the normal group receiving LPS or in hyperglycemia animals, suggesting that LPS did not play a role in promoting apoptosis in the hyperglycemia model.

## 5 Conclusion

Taken together, LPS in such lower dosage can promote a continuous increase in urinary protein in the hyperglycemia model, which is mainly related to the damage to glomerular podocyte function in the renal cortex and the stimulation of the inflammatory factor expression in the renal medulla. This work provides a pathophysiological basis for LPS to synergize with diabetic kidney injury and this finding is helpful not only for making stable urinary protein animal models as well as for the research and development of antidiabetic kidney injury drugs.

## Supporting information

S1 Data (ZIP)
