# Supplementary material for: Low-dose lipopolysaccharide inducing continuous and obvious increase in urinary protein in hyperglycemic rats and the underlying mechanism
Source: PLoS One. 2023 Jul 19;18(7):e0288876. doi: 10.1371/journal.pone.0288876 (PMC10355451; doi:10.1371/journal.pone.0288876)
Supplement: S1 Data — (ZIP) [file pone.0288876.s001.zip › source data/Protein original unedited and uncropped images.pdf]

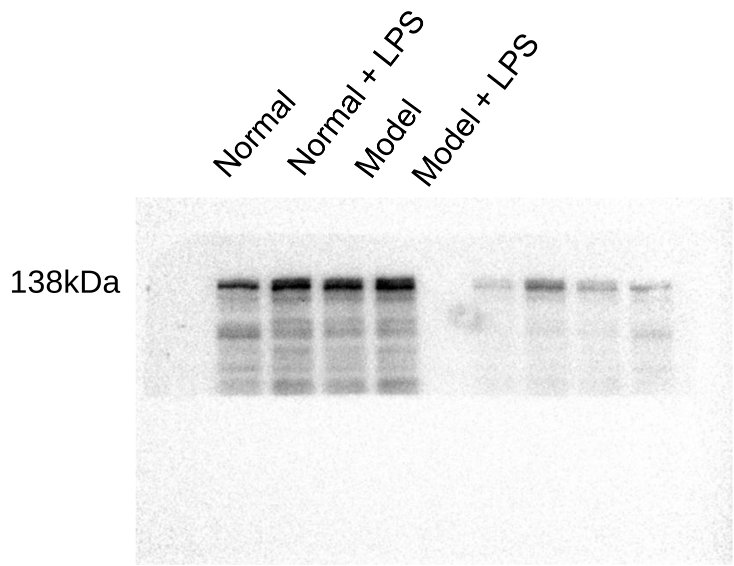


Nephrin

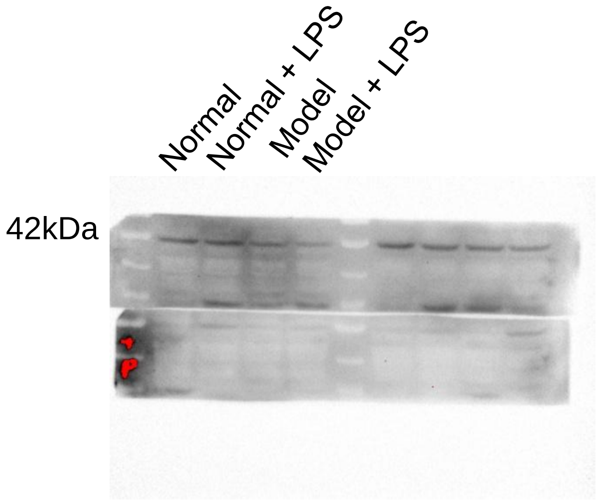


Podocin

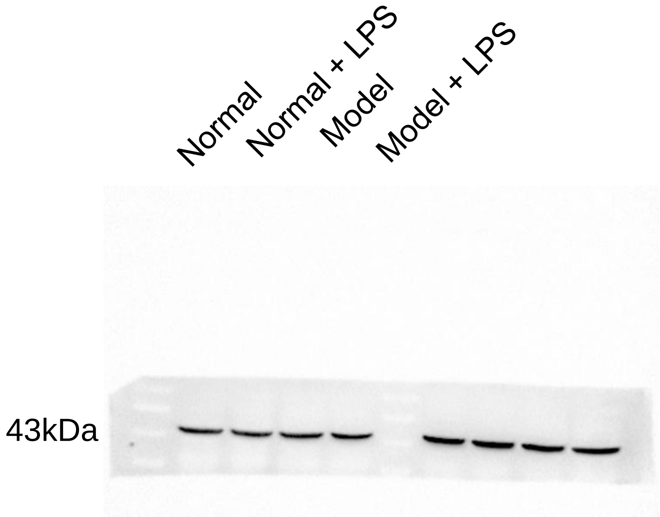


β-actin

**Figure 4** C&D& β-actin
The left four strips in the three images are used in the manuscript.

43kDa

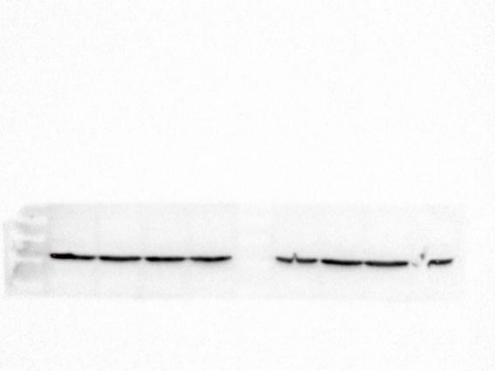

**β-actin** The left four strips are the protein in renal cortex(Fig 5 & Fig 7A);

118kDa

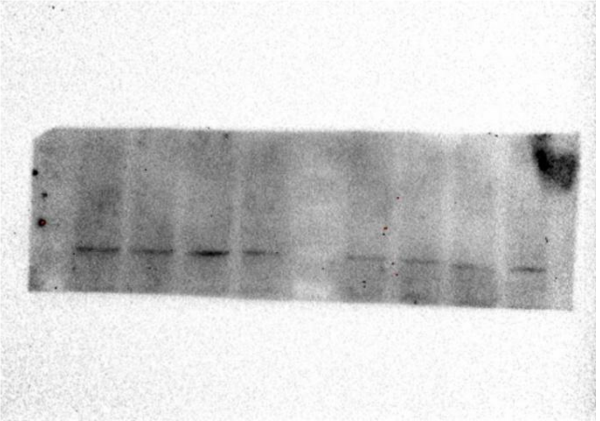

**Nlrp3** The left four strips are the protein in renal cortex(Fig5); the right four strips are the protein in renal medulla(Fig6)

22kDa

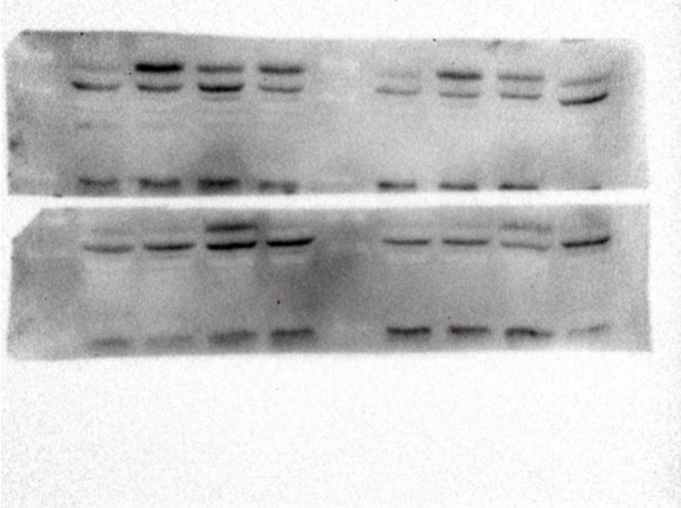

**IL-18** The lower left four bands are the protein in renal cortex(Fig 5); the lower right four strips are the protein in renal medulla(Fig 6)

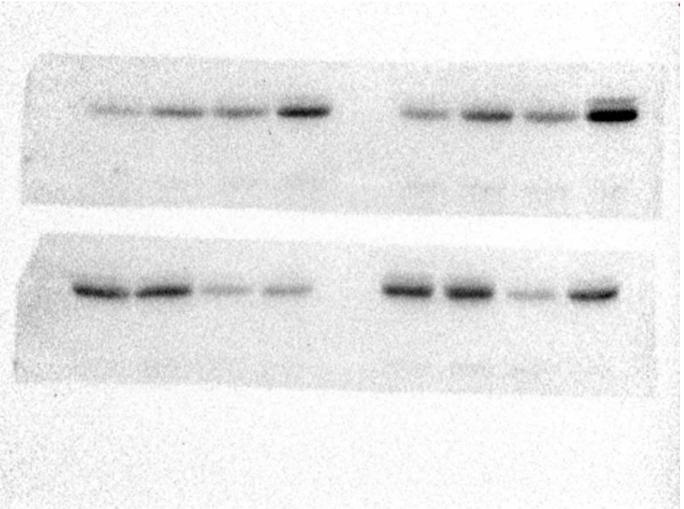

**IL-6** The lower left four bands are the protein in renal cortex(Fig 5); the lower right four strips are the protein in renal medulla(Fig 6)

45kDa

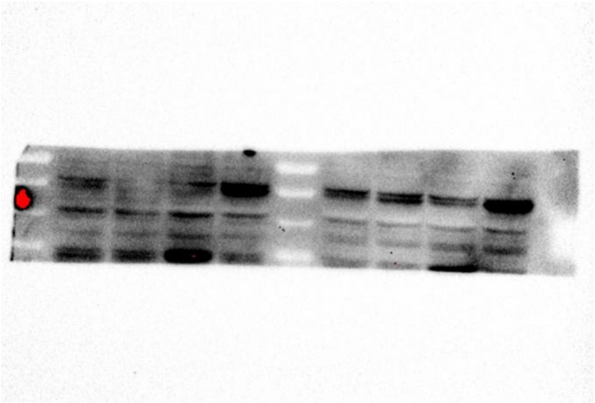

**Caspase-1** The left four strips are the protein in renal cortex(Fig 5);
the right four strips are the protein in renal medulla(Fig 6)

65kDa

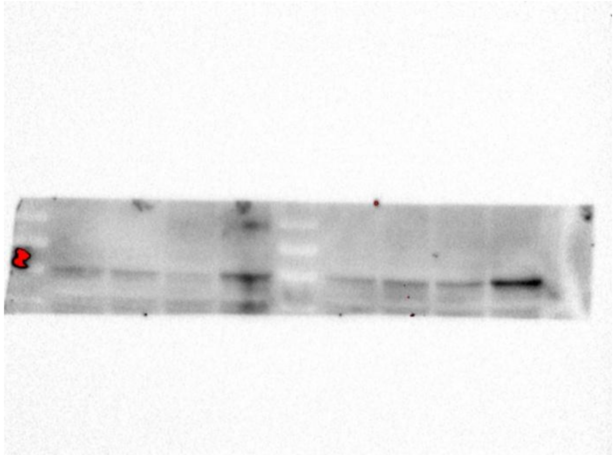

**NF-KB** The left four strips are the protein in renal cortex(Fig 5);
the right four strips are the protein in renal medulla(Fig 6)

43kDa

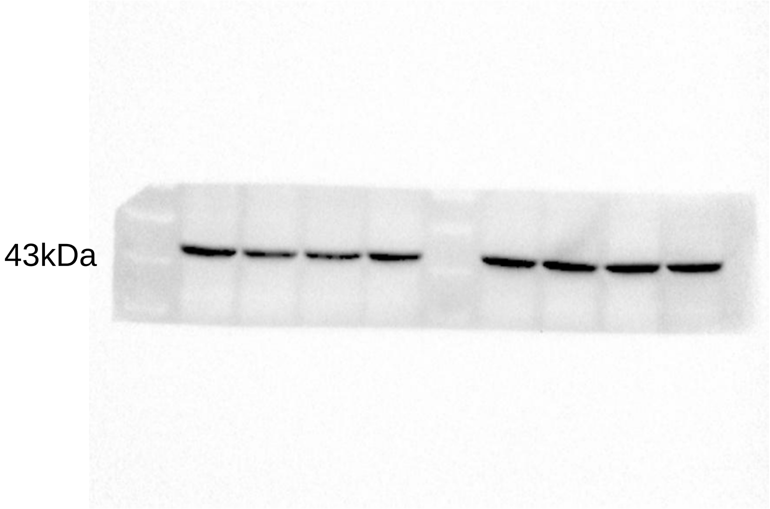

**β-actin** the right four strips are the protein in renal medulla(Fig 6 & Fig 7D)

69kDa

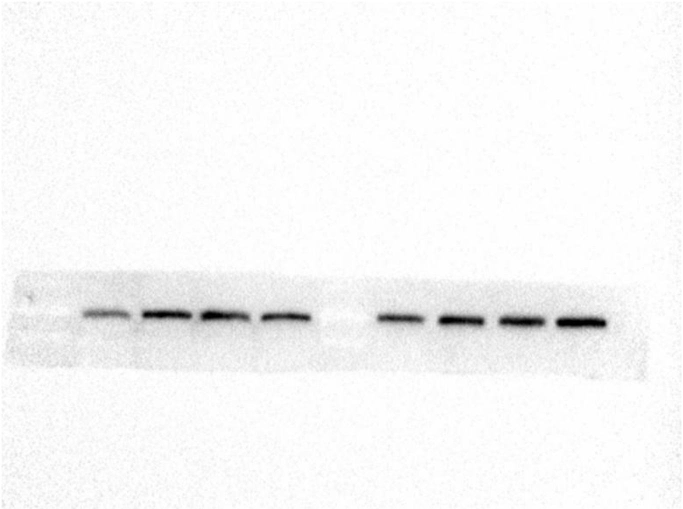

**ALB** The left four strips are the protein in renal cortex (Fig 7A);
the right four strips are the protein in renal medulla(Fig 7D)

31kDa

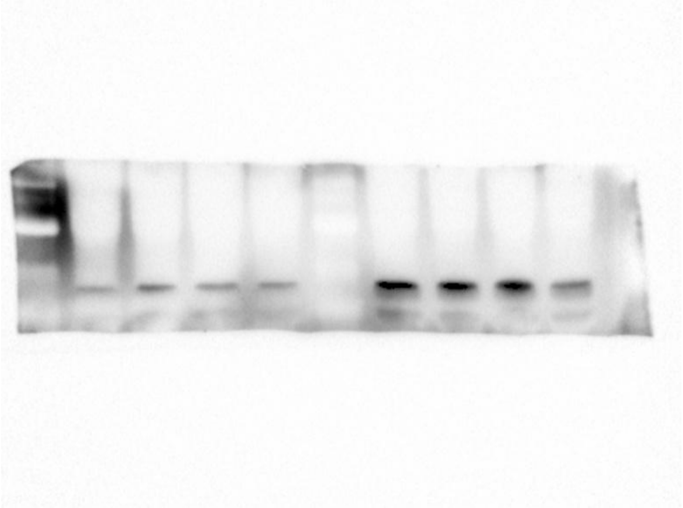

**Caspase-3** The left four strips are the protein in renal cortex (Fig 7A);
the right four strips are the protein in renal medulla (Fig 7D)

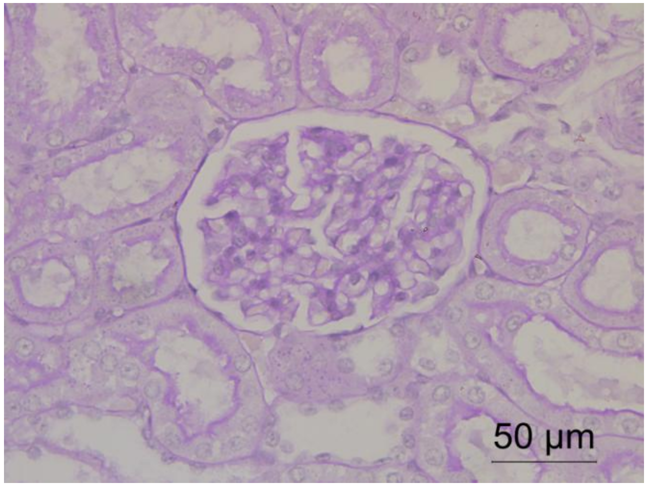


**Normal** – renal cortex

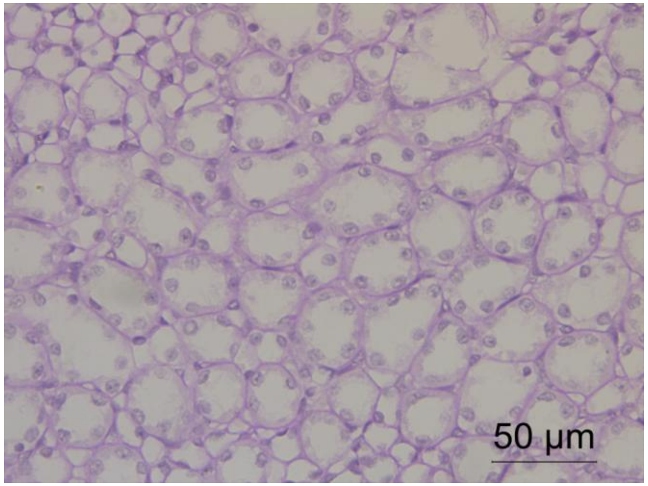


**Normal** – renal medulla

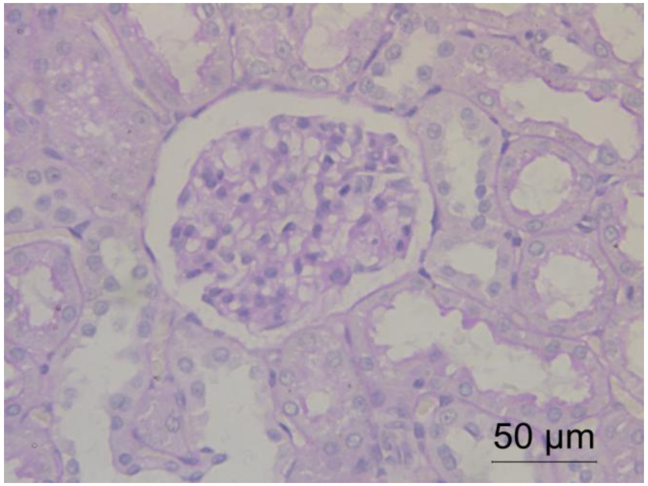


**Normal + LPS**– renal cortex

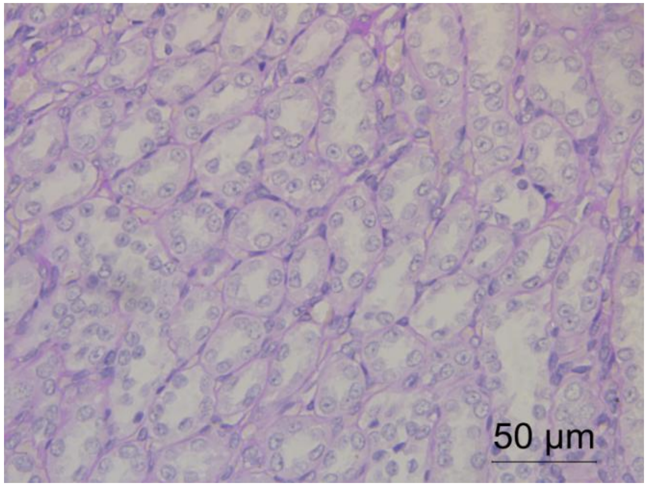


**Normal + LPS** – renal medulla

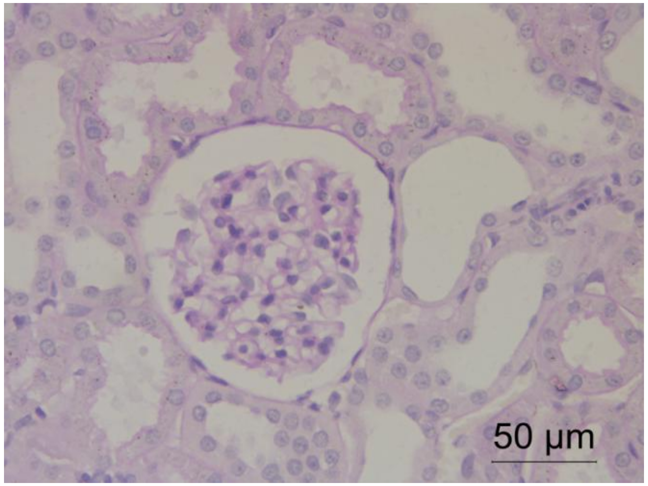


**Model** – renal cortex

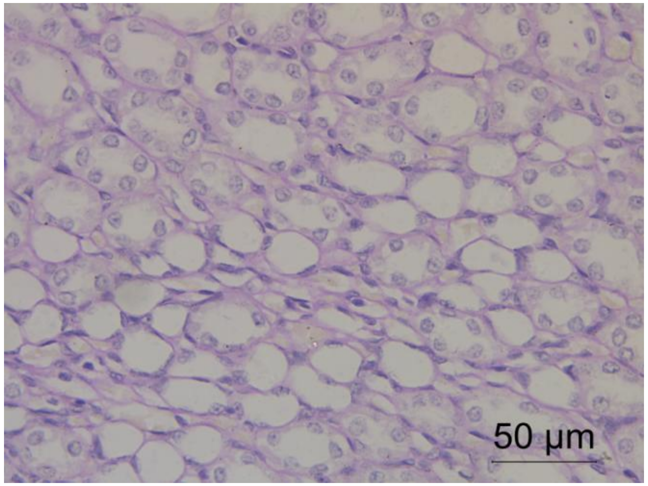


**Model** – renal medulla

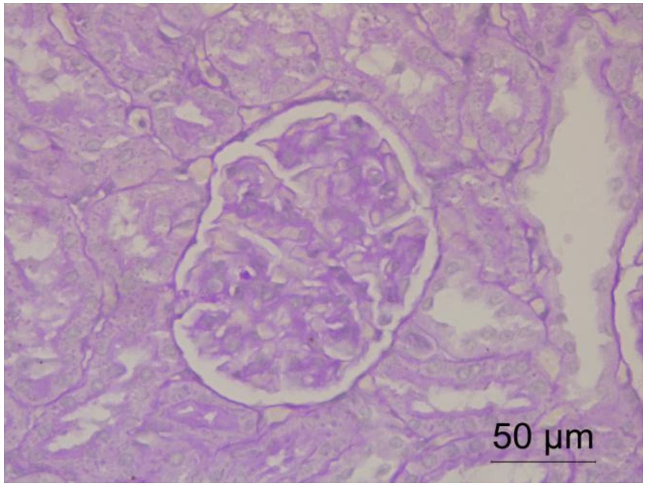


**Model + LPS** – renal cortex

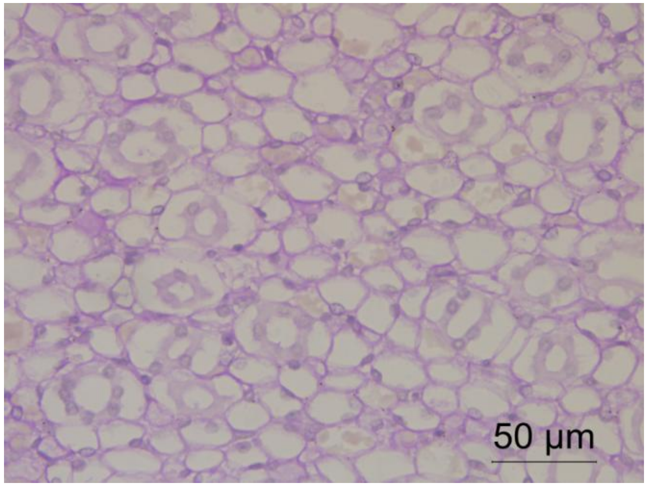


**Model + LPS** – renal medulla
